# Interplay between Maternal and Neonatal Vitamin D Deficiency and Vitamin-D-Related Gene Polymorphism with Neonatal Birth Anthropometry

**DOI:** 10.3390/nu14030564

**Published:** 2022-01-27

**Authors:** Siew Siew Lee, King Hwa Ling, Maiza Tusimin, Raman Subramaniam, Kartini Farah Rahim, Su Peng Loh

**Affiliations:** 1 Department of Nutrition, Faculty of Medicine and Health Sciences, Universiti Putra Malaysia, Serdang 43400, Malaysia; Lee.Siew@nottingham.edu.my; 2 School of Biosciences, Faculty of Science and Engineering, University of Nottingham Malaysia, Semenyih 43500, Malaysia; 3 Department of Biomedical Sciences, Faculty of Medicine and Health Sciences, Universiti Putra Malaysia, Serdang 43400, Malaysia; lkh@upm.edu.my; 4 Prince Court Medical Centre, Kuala Lumpur 50450, Malaysia; drmaizatusimin@gmail.com; 5 Fetal Medicine and Gynaecology Centre (FMGC), Petaling Jaya 46200, Malaysia; raman1321951@gmail.com; 6 Avisena Specialist Hospital, Shah Alam 40000, Malaysia; drkartinirahim@gmail.com; 7 Research Centre of Excellence for Nutrition and Non-Communicable Diseases, Faculty of Medicine and Health Sciences, Universiti Putra Malaysia, Serdang 43400, Malaysia

**Keywords:** vitamin D deficiency, single nucleotide polymorphism, birth weight, head circumference, crown–heel length

## Abstract

Vitamin D deficiency during pregnancy has been associated with poor foetal growth and neonatal birth anthropometry. However, the associations were inconsistent and could be confounded by neonatal vitamin D status and genetic factors. Until recently, limited studies have concomitantly examined the effect of maternal and neonatal vitamin D deficiency and vitamin D-related single nucleotide polymorphisms (SNPs) on neonatal birth anthropometry. This study aims to examine the independent and combined effects of maternal and neonatal vitamin D deficiency and vitamin-D-related SNPs on neonatal birth anthropometry. This cross-sectional study included 217 mother–neonate dyads recruited from Hospital Serdang, Selangor, Malaysia, between 2015 and 2017. Plasma 25-hydroxyvitamin D (25OHD) concentration was measured in maternal and umbilical cord blood using ultra-high-performance liquid chromatography (UHPLC). Maternal and neonatal vitamin D Receptor (VDR) SNP (rs2228570) genotypes were determined using high-resolution melting (HRM). Group-specific component (GC) SNPs (rs4588 and rs7041) genotypes were determined using restriction fragment length polymorphism. Our results showed that: (1) maternal vitamin D deficiency (25OHD < 30 nmol/L) was inversely associated with birth weight, head circumference and crown–heel length; (2) neonatal SNPs, *VDR* rs2228570 and *GC* rs4588, were significantly associated with birth weight and head circumference, respectively; and (3) a potential interaction was observed between maternal *VDR* rs2228570 with maternal vitamin D deficiency on head circumference. These findings suggest that the underlying mechanisms of vitamin D on foetal growth are likely to be localised in the maternal compartment, mediated through the placenta, rather than through cellular mechanisms within the foetus. Further large-scale studies are warranted to validate and extend these findings.

## 1. Introduction

Within the paradigm of developmental origins of health and disease, the intrauterine environment plays an important role in programming the risk of disease later in life [1,2]. A poor intrauterine nutrition environment leads to poor foetal growth and development, resulting in poor neonatal anthropometry at birth. Birth weight, head circumference and crown–heel length are measurements that are routinely measured and recorded soon after the birth of the newborn. These measurements, the indicators of size at birth, provide important information on the foetal intrauterine nutritional and developmental status [3] and have been associated with morbidity and development of disease later in life [1,2]. For example, birth weight has been associated with adult cardiometabolic disease [2,4], and head circumference has been associated with risk for neuropsychiatric disorders of developmental origins [5].

Vitamin D, a secosteroid hormone, has important functions in calcium homeostasis and bone metabolism. Plasma or serum 25-hydroxyvitamin D (25OHD) has been widely used as a biomarker of vitamin D status [6,7], and in circulation, about 80–90% of the 25OHD is bound to vitamin D binding protein (VDBP) and about 12% is bound to albumin, leaving less than 1 percent present as free form [8]. Recently, vitamin D has been depicted to have extra-skeletal roles related to foetal growth, including cell proliferation [9], adipogenesis [10], immunomodulation [11] and glucose homeostasis [9,12]. Vitamin D deficiency in pregnant women can affect foetal growth and anthropometric parameters at birth.

Observational studies have shown mixed results on the associations between maternal 25OHD concentrations and neonatal birth anthropometry. While several studies have reported positive linear associations between maternal 25OHD concentrations with birth weight [13,14,15,16], Gernand et al. [17] demonstrated that the association was non-linear and levelled off at 25OHD greater than 37.5 nmol/L. In a large nested case–control study in Beijing, China, low maternal 25OHD concentrations were associated with a higher birth weight and an increased risk of macrosomia [18,19]. Several observational studies did not observe a significant association between maternal 25OHD concentrations and birth weight [20,21,22,23]. In a recent meta-analysis of observational studies, vitamin-D-deficient mothers, defined as 25OHD concentrations < 30 nmol/L, had offspring with a lower birth weight and head circumference and a higher risk of being small for gestational age (SGA) [24]. In the meta-analyses of randomised controlled trials, maternal supplementation was demonstrated to have a significant positive effect on birth weight, head circumference and length at birth in the pooled analysis of 10, 5 and 3 trials studies, respectively [25]. The evidence on vitamin D and birth size remained weak, which the quality of evidence varied from very low to moderate, and the effect of vitamin D on birth size could be confounded by several factors, including genetics factors, maternal 25OHD levels at baseline and neonatal vitamin D status [25].

Despite the growing body of evidence on the association between maternal vitamin D deficiency and neonatal birth anthropometry, the mechanism underlying the association remains to be explored. There are many plausible ways for maternal vitamin D deficiency to impact neonatal birth anthropometry. Maternal vitamin D deficiency can impact foetal growth directly or indirectly through reducing foetal 25OHD availability. Previous studies that examined the associations assessed either maternal or neonatal 25OHD concentrations. Given that maternal and neonatal 25OHD concentrations are highly correlated (correlations coefficients ranged from 0.3 to 0.9) [26], both variables could confound each other for their effect on birth anthropometry. Polymorphism in vitamin-D-related genes has been related to several non-skeletal health outcomes for which low 25OHD concentration is a risk factor [27], but the evidence for birth anthropometry is limited and inconsistent. Early studies by Swamy et al. [28] and Bodnar et al. [29] demonstrated no association between *VDR* rs2228570 with birth weight and SGA, respectively. In a more recent study, Barchitta et al. [30] reported a significant association between maternal *VDR* rs228570 with birth weight. In other studies, vitamin-D-related SNPs were shown to interact or confound the effect of maternal vitamin D deficiency on birth anthropometry [31,32]. Although inconsistent, recent studies have also demonstrated that bioavailable 25OHD (albumin-bound 25OHD plus free 25OHD) was better correlated with calciotropic and non-calciotropic outcomes than total 25OHD [33]. This suggests that the bioavailable 25OHD rather than the total 25OHD may be better associated with anthropometry at birth. However, limited studies have concomitantly examined the associations. Therefore, the present study aims to firstly examine the independent associations of maternal and neonatal vitamin D deficiency with neonatal birth anthropometry, and secondly explore the individual and combined effects of maternal and neonatal vitamin-D-related SNPs and vitamin D deficiency on neonatal birth anthropometry.

## 2. Materials and Methods

### 2.1. Study Design and Participants

Data were analysed from a cross-sectional study that examined the risk factors of maternal vitamin D deficiency [34]. The detailed information on the study design and maternal vitamin D deficiency prevalence has been published elsewhere [34]. Pregnant women admitted for delivery were recruited from the labour suite of the Department of Obstetrics and Gynaecology, Hospital Serdang, Selangor, Malaysia. The inclusion criteria were full-term pregnancy (gestational week ≥ 37 weeks) and singleton live birth. Maternal exclusion criteria were pre-existing diabetes mellitus, hyperthyroidism, osteomalacia, rheumatoid arthritis, hyperparathyroidism, gestational diabetes, preeclampsia, pregnancy-induced hypertension, and having had a history of bone and renal disorders. The neonatal exclusion criterion was the presence of congenital anomalies. This study was approved by the Medical Research and Ethics Committee of the Ministry of Health Malaysia (MREC) (NMRR ID: 15-786-24865).

### 2.2. Data Collection

The following data were obtained from the interview-administered questionnaire: educational level, ethnicity and vitamin D supplementation during pregnancy. Information on maternal height, pre-pregnancy weight and weight at birth for pre-pregnancy body mass index (BMI) and gestational weight gain calculations, last menstrual period (LMP), and parity were drawn from the electronic medical record and antenatal records. Gestational age at birth was determined by LMP and confirmed by the first dating scan. Birth data included neonatal date of birth, sex, birth weight, head circumference and crown–heel length were extracted from Child Health Record Book.

### 2.3. Analysis of Plasma 25OHD and Maternal Bioavailable 25OHD

Blood samples were collected from pregnant women before delivery and mixed arterial/venous umbilical cord after delivery. All collected blood samples were processed within the collection day and plasma was stored at −80 °C until analysis. Plasma 25OHD concentrations were analysed using an in-house validated ultra-high-performance liquid chromatography (UHPLC) method as described earlier [35]. The method has an inter-assay coefficient of variation (CV) of 6–7%. Maternal and neonatal vitamin D deficiency was defined as plasma 25OHD <30 nmol/L, a cut-off proposed by the National Academy of Medicine (formerly known as Institute of Medicine), which is associated with an increased risk of rickets, impaired fractional calcium absorption and decreased bone mineral content [6].

Plasma albumin was measured by using an immunoturbidimetric assay (Roche Cobas c311, Mannheim, Germany). VDBP was measured using the Quantikine Human Vitamin D Binding Protein Immunoassay Kit (R&D Systems, Minneapolis, MN, USA), following the manufacturer’s protocol. The manufacturer claimed the mean CV for the VDBP assay was 6%. The concentration of free 25OHD was calculated by using the equation from Powe et al. [36] (Equation (1)). The affinity binding constants for 25OHD with albumin and VDBP used were 6 × 10^5^ M^−1^ and 7 × 10^8^ M^−1^, respectively. Bioavailable 25OHD was calculated as the sum of free 25OHD and albumin-bound 25OHD using Equation (2) [37].

(1) $$\mathrm{Free}\;25\mathrm{OHD}=\frac{\mathrm{Total}\;25\mathrm{OHD}}{1+(6\times 10^{5}\times \mathrm{Albumin})+(7\times 10^{8}\times \mathrm{VDBP})}$$

(2) Bioavailable 25OHD=Free 25OHD+Albumin bound 25OHD 

### 2.4. Genotyping

DNA was extracted from the buffy coat using the QIAamp DNA Blood Mini Kit (Catalog no. 51106, QIAGEN, Hilden, Germany), following the manufacturer’s protocol. Based on the previous literature, three SNPs, *VDR* rs2228570, *GC* rs7041 and *GC* rs4588 were selected. They were well-studied missense SNPs (functional SNPs), whose variation in a nucleotide resulted in the change in amino acid and protein structure and/or function: *VDR* rs2228570, A-to-G allele transversion creates an alternative translation start site, resulting in a VDR protein that is 3 amino acids shorter than the wild type; *GC* rs7041, A-to-C allele transversion causes the change of amino acid aspartic acid (CT**A**) to glutamic acid (CT**C**); rs4588, G-to-T allele transversion causes the change of amino acid threonine (T**G**C) to lysine (T**T**C). These SNPs have been frequently reported to be associated with 25OHD concentrations [27,38,39] and non-skeletal health outcomes [27] in genome-wide associations studies (GWAS). Likewise, these SNPs are shared among the study population, which have minor allele frequency (MAF) of ≥20% in East Asian (EAS) and South Asian (SAS) in 1000 Genome Project [40].

The restriction fragment length polymorphism (RFLP) method was performed to determine the genotypes of rs4588 and rs7041 [34]. In brief, a PCR was performed using a thermocycler (Thermo Fisher Scientific, Waltham, MA, USA), and the PCR product, a 483-bp fragment, was then digested separately using restriction enzymes, *HaeIII* (for rs7041) and *StyI* (for rs4588) (New England Biolabs Inc., Ipswich, MA, USA). The digested products were then evaluated by electrophoresis on 1.5% agarose gel stained with ethidium bromide. The genotypes of rs2228570 were determined using LightCycler® 480 High-Resolution Melting Master (Product no. 04909631001, Roche Diagnostics, Basel, Switzerland), performed on the LightCycler 480 Instrument (Roche Diagnostics, Basel, Switzerland). Melting curve analysis was performed for genotyping: the heterozygous genotype was displayed with a difference in the shape of the melting curve, whereas changes in melting temperature discriminated the wild type and the mutant genotypes.

### 2.5. Statistical Analysis

Statistical analysis was performed using SPSS version 26.0 (SPSS Inc., Chicago, IL, USA). Hardy–Weinberg Equilibrium (HWE) for each SNP was examined using the chi-square test.

Birth weight, head circumference and crown–heel length were the three neonatal birth anthropometries examined in the study. The associations between maternal and neonatal plasma 25OHD and SNPs with these outcomes were analysed using uni- and multivariate general linear models (GLM). The associations were performed in four analyses, as follows: (1) the separate association between maternal and neonatal vitamin D deficiency and maternal bioavailable 25OHD with the birth anthropometry; (2) the separate association of maternal and neonatal *VDR* rs2228570, *GC* rs4588 and *GC* rs7041 with birth outcomes; (3) the interactions of maternal and neonatal vitamin D deficiency and SNPs by including the interaction term in the adjusted GLM models; and (4) the combined effect of maternal and neonatal vitamin D deficiency and SNPs on the birth anthropometry. To determine the combined effect, variables that had a *p*-value < 0.25 in the group-specific associations were included as a base model. The backward stepwise method was used to generate the final model of factors associated with the birth anthropometry. When required, the data and *p*-values were adjusted for maternal pre-pregnancy BMI, infant sex, gestational weight gain, parity, and maternal height.

## 3. Results

### 3.1. Maternal and Neonatal Characteristics and SNP Genotype Distributions

This study included a total of 217 mother–neonate dyads. The mean maternal age at delivery was 28.9 ± 4.2 years, and the mean pre-pregnancy maternal BMI was 23.7 ± 5.1 kg/m^2^ (Table 1). Half of the pregnant women (50.2%) were vitamin D deficient (25OHD < 30 nmol/L). The proportion of male (49.3%) and female infants (50.7%) was equal. The mean gestational age at birth for the neonate was 39.1 ± 1.1 weeks. Table 2 presents the distributions of maternal and neonatal *VDR* and *GC* SNP. The genotype distributions for all the SNPs are within Hardy–Weinberg equilibrium (χ^2^ < 0.3841, *p* > 0.05).

### 3.2. Associations of Vitamin D Deficiency and Bioavailable 25OHD with Birth Outcomes

Maternal vitamin D deficiency (plasma 25OHD < 30 nmol/L) was associated with birth weights, head circumference and crown–heel length at birth after adjustment (Table 3). Low maternal bioavailable 25OHD was significantly associated with an increase in head circumference. No significant associations were found between neonatal vitamin D deficiency and all the birth anthropometry in univariate and multivariate analyses.

### 3.3. Associations of VDR and GC SNPs with Birth Outcomes

The association of neonatal *VDR* rs2228570 with birth weight was near significant (*p* = 0.064) (Table 4). There was no significant association between two *GC* SNPs and birth weight. After adjustment, neonatal *GC* rs4588 was significantly associated with head circumference; heterozygous for neonatal *GC* rs4588 was significantly associated with 4.5 mm (95% CI: 1.1, 8.0, *p* = 0.010) larger in head circumference than other genotypes. In multivariate analyses, no significant associations were found between maternal and neonatal SNPs with crown–heel length.

### 3.4. Interaction and Combined Associations of Vitamin D Status and SNPs on Birth Outcomes

The results on the interactions of SNPs with vitamin D deficiency on birth anthropometry are available in the Online Supplementary Materials (Tables S1–S6). A potential interaction was observed between maternal *VDR* rs2228570 and maternal vitamin D deficiency on head circumferences (Table S3).

Table 5 presents the combined effect of maternal and neonatal vitamin D deficiency and SNPs on birth weight. Maternal vitamin D deficiency and cord *VDR* rs2228570 were significantly associated with birth weight. Mothers with 25OHD < 30 nmol/L gave birth to a baby with a birth weight 107 g heavier than mothers with 25OHD ≥ 30 nmol/L. Neonatal *VDR* rs2228570 “G” allele carrier was associated with a 137 g higher birth than that of the non-carrier. Together with other variables, maternal vitamin D deficiency and neonatal *VDR* rs2228570 explained a 27% birth weight variation.

After the adjustment, maternal vitamin D deficiency and low maternal bioavailable 25OHD (<1.6 μmol/L) were significantly associated with head circumference (Table 6). In addition, heterozygous for cord *GC* rs4588 was associated with a 4 mm larger head circumference than other genotypes. The head circumference of neonates who had mothers with the *VDR* rs2228570 GG genotype and maternal 25OHD <30 nmol/L was significantly higher than that of neonates who had other genotypes (AA or AG). There was no significant difference in head circumference in neonates whose maternal 25OHD concentrations were ≥30 nmol/L when compared according to maternal SNPs. 

In the final model, maternal vitamin D deficiency was significantly associated with crown–heel length, whereas maternal and neonatal SNPs examined were not associated with crown–heel length (Table 7).

## 4. Discussion

This study examined the individual and combined effect of maternal and neonatal vitamin D deficiency and vitamin D-related SNPs (*VDR* rs2228570, *GC* rs4588 and *GC* rs7041) with birth outcomes. This study showed a consistent pattern effect of vitamin D deficiency and SNPs on birth anthropometry: (1) maternal vitamin D deficiency (25OHD <30 nmol/L) demonstrated consistent associations on birth weight, head circumference and crown–heel length at birth in all the analyses; (2) neonatal SNPs, *VDR* rs2228570 and *GC* rs4588, demonstrated significant associations with birth weight and head circumference, respectively; and (3) a potential interaction was observed between maternal *VDR* rs2228570 with maternal vitamin D deficiency on head circumference.

Interestingly, we observed an inverse association between maternal vitamin D deficiency and birth weight, and this finding is contradicted by most of the previous studies that have shown a positive association between vitamin D and birth weight [13,14,16,31,41,42,43]. However, our finding was in agreement with a large cohort study by Wen et al. [19], which reported that low maternal 25OHD concentrations were associated with high birth weight [19]. The differences in the associations may be related to the methodological approach, which included the time-point of maternal blood sampling, the cut-off values used to define vitamin D deficiency and the analytical method used to assess 25OHD. Other possible explanations may be the heterogeneity in the study population, maternal 25OHD concentration and the interaction effect between vitamin D deficiency and other factors.

The interaction effect of maternal vitamin D deficiency with several factors, including pre-pregnancy BMI, infant sex and gestational diabetes, have been reported in previous studies. Sauder et al. [44] have demonstrated a significant interaction between 25OHD with pre-pregnancy BMI for total mass. The analysis demonstrated that an increase in 25OHD concentrations was associated with a decrease in birth weight among women with lower pre-pregnancy BMI [44]. Among women with a higher pre-pregnancy BMI, an increase in 25OHD was associated with an increase in birth weight [44]. These findings were further confirmed by Francis et al. [15]. In a study by Eggemoen et al. [23], there were sex differences in the associations between maternal 25OHD concentrations and birth outcomes, in which maternal 25OHD concentrations were inversely associated with the sum of skinfolds in males, but not in females. In a recent study by Liu et al. [45], severely deficient 25OHD had a decreased risk of delivering large for gestational age (LGA) infants. However, there was an additive interaction between maternal vitamin D deficiency and gestational diabetes mellitus on LGA risk.

An inverse association was also observed between maternal vitamin D deficiency and head circumference. This finding agrees with previous studies [20,46] that mothers with low vitamin D status have infants with a bigger head circumference. While most of the previous studies showed neither maternal nor cord vitamin D deficiency or 25OHD concentrations was associated with length at birth [13,15,21,41,47], the finding of our study is at odds with the previous. In addition to differences in the study design and variable control in the multivariate model, the discrepancy can be attributed to the high cut-off (50 nmol/L or 75 nmol/L) defining vitamin D deficiency in the previous study [13,15,21,41,47]. The effect of maternal vitamin D with crown–heel length at birth is potentially through the role of vitamin D in bone mineralisation. It has been shown that 25OHD concentration of <30 nmol/L was at an increased risk of vitamin D deficiency based on the role of vitamin D in bone mineralisation [6]. Hence, a cut-off higher than 30 nmol/L may not observe significant differences in outcomes related to bone mineralization, such as the crown–heel length. In support of this, the study by Miliku et al. [43] reported a significant association between maternal vitamin D deficiency, defined as 25OHD <25 nmol/L and length at birth. In a recent study, Karras et al. [48] demonstrated that maternal TAQI VDR polymorphism significantly affects neonatal birth anthropometry when maternal 25OHD were <50 nmol/L, but not for a higher cut-off of >50 nmol/L.

In the present study, the cord but not maternal *VDR* rs2228570 G allele was associated with an increased birth weight by 137 g. This finding partially agrees with a study by Barchitta et al. [30], which reported that an increased number of G alleles in maternal *VDR* SNPs was associated with a decreased birth weight. However, neonatal VDR rs2228570, which might confound the associations observed, was not assessed and adjusted in that study Barchitta et al. [30]. The confounding effect is observed in the present study, in which both maternal and neonatal *VDR* rs2228570 were associated with birth weight in the crude model. However, the association of maternal *VDR* rs2229570 with birth weight was attenuated when adjusted for neonatal *VDR* rs2228570. The associations of *VDR* rs2228570 G allele with birth weight can be explained by G allele is a mutant allele for rs2228570, which creates an alternative translation start site for the VDR gene. This results in a VDR protein that is three amino acids shorter than the wild-type allele. The short VDR isoform may be associated with increased transcriptional activity [49] related to birth weight. Further research is required to explore the possible mechanism.

Despite the growing evidence on the effect of vitamin D on foetal growth, the mechanistic pathways remain to be explored. By its classical role in calcium homeostasis and bone mineralisation, maternal vitamin D deficiency affects foetal growth by reducing foetal 25OHD availability and decreasing the local generation of 1,25(OH)_2_D in the foetus. If this is the case, results would demonstrate neonatal vitamin D deficiency is more related to birth anthropometry. Interestingly, we observed consistent maternal, but not neonatal, vitamin D deficiency associations with birth anthropometry. These findings suggest that the putative mechanism of vitamin D on foetal growth might not occur through the local role of vitamin D in the foetus in bone mineralisation. In support of this, it has been shown that the developing foetus does not require 1,25(OH)_2_D for bone development and mineral homeostasis as the foetus completely depends on the maternal transfer of calcium [50].

Previous studies have demonstrated the potential interaction effect between neonatal *VDR* rs2228570 [32] and maternal *GC* rs7041 [31] with vitamin D deficiency on birth weight. However, we found no interaction between SNPs with maternal and cord vitamin D deficiency on birth weight. This discrepancy may be explained by the difference in genotype distribution between the studies participants. For example, in the study by Chun et al. [31], more participants (53%) had TT genotype than our study (41%). Additionally, the discrepancy might reflect the lack of statistical power in our study. Our study is the first to report on the combined effects of maternal and neonatal VDR and GC polymorphism on neonatal birth outcomes. The findings further our understanding on the effect of vitamin D and foetal growth. The associations of SNPs combined with vitamin D deficiency (Figure S1 in the online Supplementary Materials) suggest that the underlying mechanisms of vitamin D on foetal growth are likely localized in the maternal compartment, mediated through the placenta, rather than through cellular mechanisms within the foetus. Vitamin D has been shown to regulate gene expression and placental hormone secretion, such as lactogen and other hormones that affect maternal glucose and fatty acid metabolism [43]. Moreover, vitamin D may possess a growth-promoting effect in the placenta. Although inconsistent, studies have shown low 25OHD during pregnancy is associated with reduced placental development and weight [17,26,51].

This study has several limitations. First, the vitamin D level assessed at the delivery might not represent the concentrations for the entire course of pregnancy. Second, the sample size was small and not powered to show the significant interaction effect of less frequent SNPs, but it was sufficiently powered to show the interaction effect of common SNPs examined in the present study. Third, the adjustment for multiple testing was not performed. This limitation potentially increases the likelihood of a false-positive result in the bivariate and multivariate association analyses. However, the variables included in the present study were selected based on previous studies.

## 5. Conclusions

In conclusion, our findings indicate that maternal vitamin D deficiency (<30 nmol/L) was inversely associated with all the neonatal birth anthropometry, while neonatal VDRrs2228570 and GCrs4588 were significantly associated with birth weight and head circumference, respectively. There are potential interactions between maternal vitamin D deficiency and maternal *VDR* rs2228570 and head circumference. Taken together, these findings demonstrate the complex interplay between maternal and neonatal vitamin D deficiency and SNP on foetal growth and suggest that the underlying mechanisms of vitamin D on foetal growth are likely localized in the maternal compartment, mediated through the placenta, rather than through cellular mechanisms within the foetus. Further large-scale studies are warranted to validate and extend our findings and understand the onset of the inverse associations between vitamin D and birth anthropometry.

## Figures and Tables

**Table 1:** Maternal and neonatal characteristics (*n* = 217).

Characteristics	Mean ± SD or *n* (%)
Maternal	
Age (years)	28.9 ± 4.2
Ethnicity [*n* (%)]	
Malay	187 (86.2)
Chinese	20 (9.2)
Indians and others ^a^	10 (4.6)
Height (cm)	155.7 ± 5.7
Pre-pregnancy weight (kg)	57.6 ± 13.2
Pre-pregnancy body mass index (kg/m^2^)	23.7 ± 5.1
Weight at birth (kg) (*n* = 216) ^b^	68.1 ± 12.5
Gestational weight gain (kg)	10.6 ± 5.0
Education level: Secondary and lower	94 (43.3)
Nulliparous (*n* (%))	58 (26.7)
Plasma total 25OHD (nmol/L)	34.3 ± 21.2
Vitamin D deficiency (<30 nmol/L)	109 (50.2)
Bioavailable 25OHD (µmol/L)	1.8 ± 1.3
Vitamin D supplementation (*n* (%))	87 (40.1)
Neonatal	
Sex: Male (*n* (%))	107 (49.3)
Gestational age at birth (weeks)	39.1 ± 1.1
Birth weight (g)	3064.3 ± 372.3
Head circumference at birth (mm)	328.3 ± 11.9
Crown-heel length (mm)	482.9 ± 18.0
Plasma total 25OHD (nmol/L)	24.9 ± 12.2
Vitamin D deficiency (<30 nmol/L)	155 (71.4)

^a^ Others included Kadazan (*n* = 1), Bajau (*n* = 1), Suluk (*n* = 1) and mixed ethnic (*n* = 1); ^b^ Where measurements were not obtained in the full set of 217 participants, the exact number of participants for the variable is stated in brackets beside the variable name.

**Table 2:** VDR and GC single nucleotide polymorphism genotype distributions of mothers and neonates.

SNP	Maternal			Neonatal		
	*n* (%)	HWE χ^2^	HWE *p*-Value	*n* (%)	HWE χ^2^	HWE *p*-Value
*VDR* rs2228570						
AA	38 (17.5)	0.035	0.982	39 (18.0)	0.055	0.973
AG	104 (47.9)			104 (47.9)		
GG	75 (34.6)			74 (34.1)		
*GC* rs4588						
GG	135 (62.2)	1.505	0.471	134 (61.8)	1.694	0.429
GT	76 (35.0)			77 (35.5)		
TT	6 (2.8)			6 (2.8)		
*GC* rs7041						
AA	89 (41.0)	0.300	0.861	100 (46.1)	0.901	0.637
AC	97 (44.7)			99 (45.6)		
CC	31 (14.3)			18 (8.3)		

**Table 3:** Associations of maternal and neonatal vitamin D deficiency and maternal bioavailable 25OHD with birth outcomes ^a^.

Variables	Birth Weight (g)						Head Circumference (mm)						Crown–Heel Length (mm)					
	Crude Model			Adjusted Model ^b^			Crude Model			Adjusted Model ^b^			Crude Model			Adjusted Model ^c^		
	β	95% CI	*p*-Value	β	95% CI	*p*-Value	β	95% CI	*p*-Value	β	95% CI	*p*-Value	β	95% CI	*p*-Value	β	95% CI	*p*-Value
Maternal 25OHD																		
<30 nmol/L	100.2	1.2, 199.2	0.047	194.8	40.2, 349.3	0.014	1.2	−2.0, 4.4	0.471	6.3	0.9, 11.7	0.022	5.3	0.5, 10.1	0.030	10.8	2.8, 18.9	0.008
≥30 nmol/L	Reference			Reference			Reference			Reference			Reference			Reference		
																		
Maternal Bio 25OHD																		
Low (<1.6 μmol/L)	41.2	−58.5, 140.9	0.417	117.5	−269.0, 34.0	0.128	−1.2	−4.4, 2.0	0.460	−6.6	−11.8, −1.3	0.015	−1.9	−6.8, 2.9	0.433	6.7	−1.2, 14.5	0.096
High (≥1.6 μmol/L)	Reference			Reference			Reference			Reference			Reference			Reference		
																		
Neonatal 25OHD																		
<30 nmol/L	60.1	−50.1, 170.4	0.284	16.1	91.4, 123.7	0.768	1.1	−2.4, 4.7	0.523	0.3	−3.5, 4.0	0.885	3.0	−2.3, 8.3	0.269	1.2	−4.3, 6.7	0.659
≥30 nmol/L	Reference			Reference			Reference			Reference			Reference			Reference		

25OHD, 25-hydroxyvitamin D; Bio, bioavailable. ^a^ Data are presented as regression coefficients and confidence intervals (CI) and reflect the differences in neonatal anthropometry compared to the reference group. ^b^ Multivariate analysis included maternal vitamin D status, maternal bioavailable 25OHD and cord vitamin D status, adjusted for gestational age at delivery, infant’s sex, maternal pre-pregnancy BMI, gestational weight gain and parity. ^c^ Multivariate analysis included maternal vitamin D status, maternal bioavailable 25OHD and cord vitamin D status, adjusted for gestational age at delivery, infant’s sex, gestational weight gain, parity and maternal height. Reference indicated which category is the reference(baseline) for the given statistical analysis.

**Table 4:** Multivariate analysis of maternal and neonatal VDR and GC SNPs with birth outcomes.

SNPs	Birth Weight (g) ^a^			Head Circumference (mm) ^a^			Crown–Heel Length (mm) ^b^		
	β	95% CI	*p*-Value	β	95% CI	*p*-Value	β	95% CI	*p*-Value
Maternal *VDR* rs2228570	81.8	−38.5, 202.2	0.181	2.3	−1.3, 5.9	0.205	0.1	−4.7, 4.8	0.977
Maternal *GC* rs7041	11.9	−92.5, 116.3	0.823	−2.1	−5.7, 1.5	0.251	3.8	−1.7, 9.2	0.175
Maternal *GC* rs4588	−39.1	−141.1, 62.8	0.450	−0.4	−3.8, 3.1	0.828	0.3	−5.8, 5.1	0.904
Neonatal *VDR* rs2228570	113.0	−6.5, 232.5	0.064	−0.1	−3.7, 3.5	0.956	3.6	−1.0, 8.3	0.126
Neonatal *GC* rs7041	−52.2	−157.4, 53.1	0.330	0.7	−3.0, 4.3	0.713	4.6	0.9, 10.0	0.102
Neonatal *GC* rs4588	95.3	−8.6, 199.2	0.072	4.5	1.1, 8.0	0.010	−1.7	−7.2, 3.9	0.555

^a^ Multivariate analysis included maternal vitamin D status, maternal bioavailable 25OHD and neonatal vitamin D status, adjusted for gestational age at delivery, infant’s sex, maternal pre-pregnancy BMI, gestational weight gain and parity. ^b^ Multivariate analysis included maternal vitamin D status, maternal bioavailable 25OHD and neonatal vitamin D status, adjusted for gestational age at delivery, infant’s sex, gestational weight gain, parity and maternal height.

**Table 5:** The final model of factors associated with infant birth weight.

Variables	Beta	95% CI	*p*-Value	Total Adjusted R^2^
Parity	47.5	13.7, 81.4	0.006	0.267
GWG	21.8	12.3, 31.4	0.0001	
Pre-BMI	16.8	7.4, 26.3	0.001	
Gestational age at birth	89.3	50.2, 128.4	0.0001	
Infant’s sex	154.4	67.1, 241.6	0.001	
Maternal 25OHD < 30 nmol/L	107.4	21.4, 193.5	0.015	
Cord VDR rs2228570	137.0	24.0, 249.9	0.018	

**Table 6:** The final model of factors associated with infant head circumference at birth ^a^.

Variables	Beta	95% CI	*p*-Value	Total Adjusted R^2^
Nulliparous	5.3	1.8, 8.7	0.003	0.138
GWG	0.4	0.1, 0.8	0.013	
Pre-BMI	0.4	0.1, 0.8	0.010	
Gestational age at birth	1.6	0.3, 3.0	0.018	
Maternal 25OHD < 30 nmo/L	9.6	3.0, 16.1	0.004	
Maternal Bio 25OHD < 1.6 μmol/L	−5.6	−0.3, −10.8	0.038	
Cord GC rs4588 (GT vs. GG + TT)	3.5	0.3, 6.7	0.031	
Interaction of M VD status and M VDR rs2228570				
25OHD < 30 nmol/L and VDR GG	5.1	0.6, 9.6	0.028	
25OHD < 30 nmol/L and VDR non-GG	Reference			
25OHD ≥ 30 nmol/L and VDR GG	−0.8	−5.2, 3.6	0.719	
25OHD ≥ 30 nmol/L and VDR non-GG	Reference			

^a^ Data are presented as regression coefficients and confidence intervals (CI) and reflect the differences in neonatal anthropometry compared to the reference group for categorical variables. Reference indicated which category is the reference(baseline) for the given statistical analysis.

**Table 7:** The final model of factors associated with crown–heel length at birth.

Variables	Beta	95% CI	*p*-Value	Total Adjusted R^2^
Infant’s sex (Boy)	6.3	1.8, 10.8	0.007	0.129
Gestational age at delivery	3.9	1.9, 5.9	0.0001	
Maternal height	0.6	0.2, 1.0	0.006	
Maternal 25OHD < 30 nmol/L	11.2	3.9, 19.1	0.005	
Low Maternal Bio 25OHD	6.6	−1.2, 14.4	0.099	

## Data Availability

Data are available from the corresponding author on reasonable request.

## Supplementary Materials

The following supporting information can be downloaded at: https://www.mdpi.com/article/10.3390/nu14030564/s1. Table S1: Associations between SNPs and infant birth weight according to maternal vitamin D status; Table S2: Associations between SNPs and infant birth weight according to neonatal vitamin D status; Table S3: Associations between SNPs and head circumference according to maternal vitamin D status; Table S4: Associations between SNPs and head circumference according to neonatal vitamin D status; Table S5: Associations between SNPs and crown–heel length according to maternal vitamin D status; Table S6: Associations between SNPs and crown–heel length according to neonatal vitamin D status; Figure S1: Schematic representation of the metabolism of vitamin D in mothers, placenta and foetus.
