# Supplementary material for: Interplay between Maternal and Neonatal Vitamin D Deficiency and Vitamin-D-Related Gene Polymorphism with Neonatal Birth Anthropometry
Source: Nutrients. 2022 Jan 27;14(3):564. doi: 10.3390/nu14030564 (PMC8839863; doi:10.3390/nu14030564)
Supplement: Supplementary file 1 [file nutrients-14-00564-s001.zip › nutrients-1558890-supplementary.pdf]

Table S1: Associations between SNPs and infant birth weight according to maternal vitamin D status

SNP	Maternal 25OHD	Beta	95% CI	P association	P interaction[a]
Maternal *VDR* rs2228570	<30nmol/L	-119.2	-295.1, 56.8	0.182	0.920
	≥30nmol/L	-129.8	-285.6, 26.1	0.102	
Maternal *GC* rs7041	<30nmol/L	-3.5	-136.2, 129.3	0.959	0.444
	≥30nmol/L	70.9	-54.7, 196.5	0.265	
Maternal *GC* rs4588	<30nmol/L	35.0	-146.7, 106.5	0.617	0.542
	≥30nmol/L	-20.1	-103.3, 173.2	0.753	
Cord *VDR* rs2228570	<30nmol/L	-121.8	-295.3, 51.7	0.167	0.877
	≥30nmol/L	-153.1	-306.3, 0.1	0.050	
Cord *GC* rs7041	<30nmol/L	-38.4	-175.3, 98.5	0.580	0.673
	≥30nmol/L	-8.9	-130.4, 112.6	0.885	
Cord *GC* rs4588	<30nmol/L	29.2	-110.2, 168.7	0.678	0.295
	≥30nmol/L	123.7	3.3, 244.1	0.044	

[a] p-value adjusted for gestational age at delivery, infant's sex, maternal pre-pregnancy BMI, gestational weight gain, and parity

Table S2: Associations between SNPs and infant birth weight according to neonatal vitamin D status

SNP	Neonatal 25OHD	Beta	95% CI	P association	P interaction[a]
Maternal *VDR* rs2228570	<30nmol/L	-109.4	-259.3, 40.6	0.152	0.872
	≥30nmol/L	-137.8	-319.5, 43.8	0.134	
Maternal *GC* rs7041	<30nmol/L	47.8	-65.0, 160.5	0.404	0.489
	≥30nmol/L	-24.1	-180.1, 132.0	0.758	
Maternal *GC* rs4588	<30nmol/L	12.5	-104.5, 129.5	0.833	0.856
	≥30nmol/L	-43.7	-197.7,110.3	0.572	
Cord *VDR* rs2228570	<30nmol/L	-142.7	-294.3, 8.9	0.065	0.799
	≥30nmol/L	-103.1	-276.3, 70.1	0.238	
Cord *GC* rs7041	<30nmol/L	--87.4	-202.2, 27.4	0.135	0.090
	≥30nmol/L	90.0	-59.4, 239.4	0.232	
Cord *GC* rs4588	<30nmol/L	76.0	-40.6, 192.6	0.200	0.261
	≥30nmol/L	56.5	-95.9, 208.8	0.461	

[a] p-value adjusted for gestational age at delivery, infant's sex, maternal pre-pregnancy BMI, gestational weight gain and parity

Table S3: Associations between SNPs and head circumference according to maternal vitamin D status

SNP	Maternal 25OHD	Beta	95% CI	P association	P interaction[a]
Maternal *VDR* rs2228570	<30nmol/L	5.1	0.2, 9.9	0.042	0.052
	≥30nmol/L	-1.4	-5.6, 2.8	0.513	
Maternal *GC* rs7041	<30nmol/L	-1.4	-6.2, -3.3	0.567	0.458
	≥30nmol/L	-3.5	-7.6, 0.6	0.097	
Maternal *GC* rs4588	<30nmol/L	1.8	-3.1, 6.7	0.475	0.260
	≥30nmol/L	-0.8	-5.1, 3.5	0.710	
Cord *VDR* rs2228570	<30nmol/L	1.0	-4.1, 6.1	0.704	0.712
	≥30nmol/L	0.6	-3.6, 4.7	0.794	
Cord *GC* rs7041	<30nmol/L	0.2	-4.6, 5.1	0.920	0.570
	≥30nmol/L	1.8	-2.3, 5.9	0.393	
Cord *GC* rs4588	<30nmol/L	4.0	-0.9, 8.9	0.108	0.967
	≥30nmol/L	4.2	-0.1, -8.3	0.046	

[a] p-value adjusted for gestational age at delivery, infant's sex, maternal pre-pregnancy BMI, gestational weight gain, and parity

Table S4: Associations between SNPs and head circumference according to neonatal vitamin D status

SNP	Noenatal 25OHD	Beta	95% CI	P association	P interaction[a]
Maternal *VDR* rs2228570	<30nmol/L	3.4	-0.4, 7.1	0.081	0.161
	≥30nmol/L	-2.7	-9.4, 4.0	0.428	
Maternal *GC* rs7041	<30nmol/L	-2.1	-5.8, 1.7	0.273	0.855
	≥30nmol/L	-1.8	-7.8, 4.2	0.547	
Maternal *GC* rs4588	<30nmol/L	2.0	-2.0, 6.0	0.316	0.209
	≥30nmol/L	2.2	-8.1, 3.6	0.447	
Cord *VDR* rs2228570	<30nmol/L	1.4	-2.5, 5.4	0.478	0.462
	≥30nmol/L	-1.6	-7.8, 4.7	0.619	
Cord *GC* rs7041	<30nmol/L	1.2	-2.7, 5.0	0.554	0.729
	≥30nmol/L	0.5	-5.5, 6.5	0.876	
Cord *GC* rs4588	<30nmol/L	0.3	-6.0, 6.1	0.936	0.197
	≥30nmol/L	5.3	1.4, 9.1	0.008	

[a] p-value adjusted for gestational age at delivery, infant's sex, maternal pre-pregnancy BMI, gestational weight gain, and parity

Table S5: Associations between SNPs and crown-heel length according to maternal vitamin D status

SNP	Maternal 25OHD	Beta	95% CI	P association	P interaction[a]
Maternal *VDR* rs2228570	<30nmol/L	-2.7	-8.4, 3.1	0.357	0.250
	≥30nmol/L	3.1	-4.2, 10.4	0.405	
Maternal *GC* rs7041	<30nmol/L	2.6	-3.2, 8.3	0.379	0.693
	≥30nmol/L	0.6	-7.0, 8.3	0.868	
Maternal *GC* rs4588	<30nmol/L	-2.6	-8.6, 3.3	0.382	0.384
	≥30nmol/L	1.2	-6.2, 8.7	0.746	
Cord *VDR* rs2228570	<30nmol/L	5.8	0.1, 11.5	0.046	0.435
	≥30nmol/L	1.2	-6.1, 8.6	0.739	
Cord *GC* rs7041	<30nmol/L	-1.2	-7.0, 4.6	0.689	0.148
	≥30nmol/L	6.1	-1.1, 13.2	0.095	
Cord *GC* rs4588	<30nmol/L	0.3	-5.8, 6.4	0.921	0.790
	≥30nmol/L	1.5	-8.8, 5.9	0.694	

[a] p-value adjusted for infant sex, gestational weight at birth and maternal height

Table S6: Associations between SNPs and crown-heel length according to neonatal vitamin D status

SNP	Neonatal 25OHD	Beta	95% CI	P association	P interaction[a]
Maternal *VDR* rs2228570	<30nmol/L	-0.9	-6.5, 4.6	0.739	0.301
	≥30nmol/L	4.5	-4.0, 13.1	0.292	
Maternal *GC* rs7041	<30nmol/L	1.5	-4.2, 7.1	0.606	0.732
	≥30nmol/L	3.0	-6.0, 11.9	0.509	
Maternal *GC* rs4588	<30nmol/L	-2.5	-8.3, 3.3	0.389	0.397
	≥30nmol/L	1.8	-7.0, 10.6	0.684	
Cord *VDR* rs2228570	<30nmol/L	3.1	-2.6, 8.6	0.281	0.575
	≥30nmol/L	6.2	-2.8, 15.1	0.173	
Cord *GC* rs7041	<30nmol/L	3.1	-2.5, 8.7	0.270	0.606
	≥30nmol/L	1.0	-7.8, 9.8	0.827	
Cord *GC* rs4588	<30nmol/L	0.8	-6.7, 5.0	0.776	0.858
	≥30nmol/L	0.2	-8.8, 9.2	0.963	

[a] p-value adjusted for infant sex, gestational weight at birth and maternal height

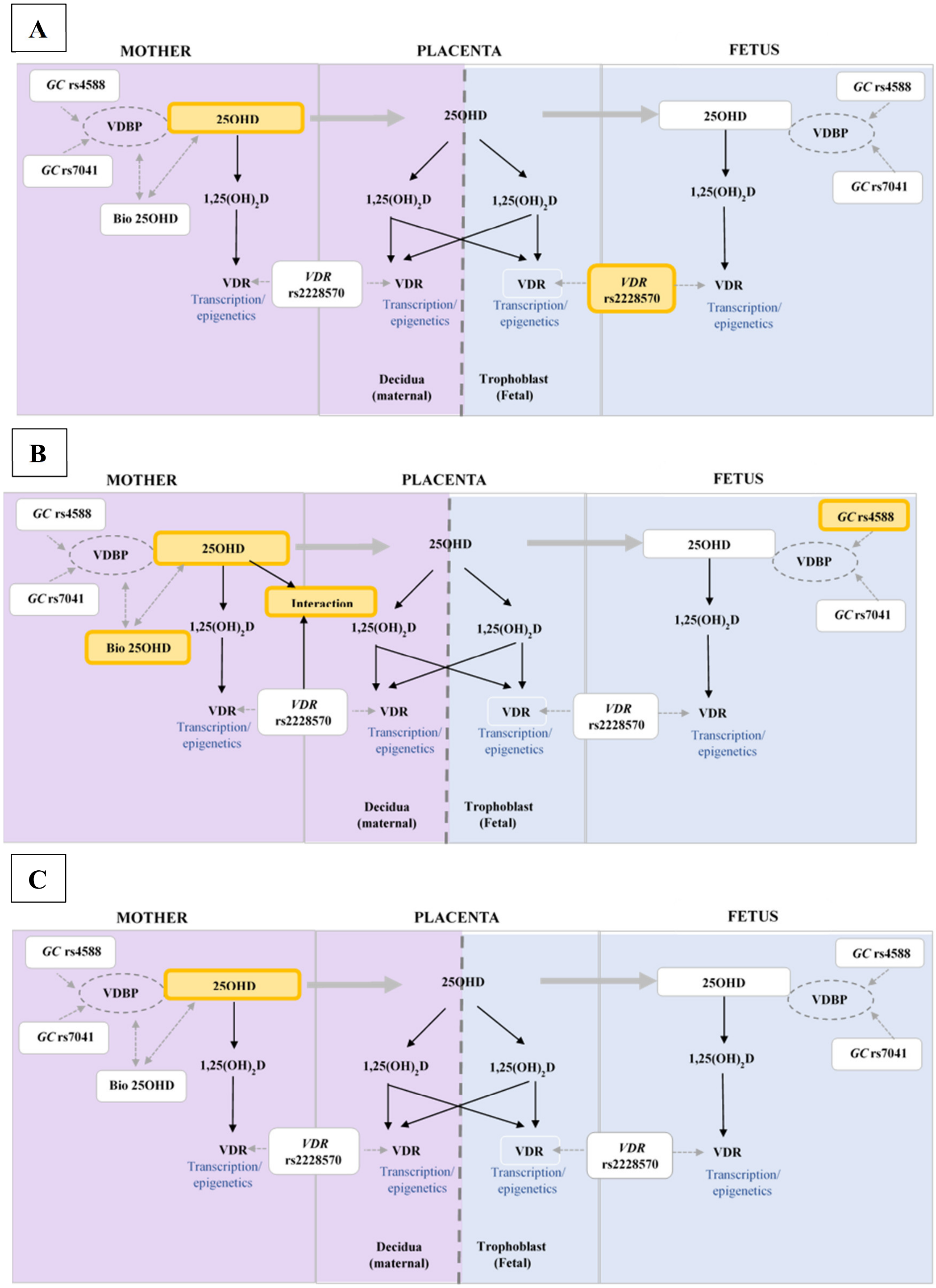

Figure S1: Schematic representation of the metabolism of vitamin D in mothers, placenta and fetus. 25-hydroxcyvitamin D (25OHD) is transferred from maternal circulation to fetus through the placenta. In fetal and placental side, 25OHD can be hydroxylated to an active form of 1,25-dihydroxyvitamin D [1,25(OH)2D], which can bind with vitamin D receptor (VDR) associated with caveolae of the plasma membrane to generate nongenomic responses, and can interact with the nuclear VDR to generate genomic responses through regulation of gene transcription or alter the epigenome. Polymorphism in VDR gene (rs2228570) could influence or modify the effect of vitamin on fetal growth. Terms in boxes represent the variables examined in this study. Term in yellow box represents significant associations with (A) birth weight, (B) Head circumference at birth (C) crown-heel length.

Term in boxes represent the variables examined in this thesis. Term in yellow box represents significant associations with (A) birth weight, (B) Head circumference at birth (C) Length at birth.

In overall, results showed that maternal vitamin D deficiency (not cord vitamin D) deficiency was associated with birth outcomes. In contrast, cord vitamin D related SNPs (not maternal SNPs) was associated with several birth outcomes.
